# Preliminary Research on the Physical and Mechanical Properties of Alternative Lightweight Aggregates Produced by Alkali-Activation of Waste Powders

**DOI:** 10.3390/ma11071255

**Published:** 2018-07-21

**Authors:** Piergiorgio Tataranni, Giulia Maria Besemer, Villiam Bortolotti, Cesare Sangiorgi

**Affiliations:** DICAM Department, University of Bologna, 40136 Bologna, Italy; giuliamaria.besemer@studio.unibo.it (G.M.B.); villiam.bortolotti@unibo.it (V.B.); cesare.sangiorgi4@unibo.it (C.S.)

**Keywords:** alkali-activated materials, lightweight aggregates, nuclear magnetic resonance, expanded clay

## Abstract

There is growing interest in construction field issues related to environmental protection, energy saving and raw materials. Therefore, the interest in recycling waste materials to produce new construction ones is constantly increasing. This study proposes a new methodology to produce lightweight aggregates (LWAs) by alkali-activation of two different waste powders: a digested spent bentonite clay and a basalt powder. Metakaolin, as secondary precursor, was added to the mixtures according to mix-design proportions, to improve the mechanical properties of the final materials, while a specific activators mix of Sodium Silicate and Sodium Hydroxide enabled the alkali-activation. The expansion process, on the other hand, was obtained using Peroxide within the liquid mix. The experimental LWAs were analyzed and tested in compliance with the EN 13055-1 standard. A more in-depth analysis on LWAs’ air voids content and porosity was also carried out by the means of Mercury Intrusion Porosimetry and Nuclear Magnetic Resonance. The results were compared with those obtained from commercial Lightweight Expanded Clay Aggregate, which represents one of the most common LWAs in the construction field. According to the presented preliminary results, the use of alkali-activated waste powders seems to be a suitable solution for the production of eco-friendly LWAs by allowing the recycling of waste materials and energy saving for their production.

## 1. Introduction and Background

Light Weight Aggregates (LWAs) are granular materials characterized by high porosity and low density. The European Standard EN 13055-1 [1] refers to LWAs as construction materials which present bulk density values lower than 1.2 Mg/m^3^ and particle density values lower than 2.0 Mg/m^3^. When used in the construction industry, LWAs offer functional and economic advantages. The pores of these materials determine favorable thermal and acoustical insulation properties and permit an overall reduction of the self-weight of the final products. LWAs can be found in nature, but most commonly they are artificially produced, either from natural raw materials or from industrial by-products [2]. In the latter case, source materials are subjected to specific treatments, which can be set to control the physical and microstructural properties of the resulting artificial LWA. The most widely used artificial LWAs are based on the processing of clay, slate, perlite, vermiculite, waste glass and ash. Among these, the use of Lightweight Expanded Clay Aggregate (LECA) is probably the most popular. LECA is a widely available and normed material that can be used in various applications including civil engineering projects. The production process of clay LWA comprises the pre-treatment of the raw dried clay and its consequent firing inside a rotary kiln. During the first stages of the process, the raw clay is finely grouted and formed in granules through the addition of water. The pelletized clay consequently undergoes drying and sintering at temperatures typically between 1050 °C and 1250 °C. The heating and rotating action of the rotary kiln leads to decomposition processes and the generation of gases. These are mainly released by the decomposition and reduction of ferric oxides, by the combustion of organic particles, the blowing of entrapped water molecules and the decomposition of carbonates [3]. The formed gases act like blowing agents and expand the clay matter, which swells forming granules with volumes up to five times the initial pellets’ dimensions. The granules are characterized by a porous inner structure and an outer hardened shell and result in an optimized weight-to-strength relationship. The physical and mechanical characteristics of LECA vary depending on the raw materials’ composition, the firing temperature, and the rotation speed. Materials used in construction industry can present a compressive strength up to 4.5 N/mm^2^ and a density of approximately 0.6 Mg/m^3^, with figures varying slightly according to the different producers.

Notations are listed in Table 1.

In recent years, increasing effort has been invested into finding new ways to reuse waste materials [4,5,6] to produce LWAs. The main benefits are represented by the reduction of the production impacts, due to the replacement of natural raw materials, and by the reuse of considerable volumes of wastes. LWAs produced from industrial by-products, such as fly-ash, sludge, and other more unconventional raw materials, can be obtained from similar production processes that are fundamentally based on the sintering of materials at high temperatures between 1000 °C and 1200 °C. Despite the beneficial incorporation and reuse of waste materials in LWAs, the energy demand of traditional production processes still represents a detrimental fact. To meet the deeper ecological goals, the cold-bonding agglomeration process is a valuable technique to produce artificial LWAs without the need of high-temperature sintering. The process is based on the use of a sloping rotary disc, where the wetting agent is added by drops and the material is pelletized by the action of scrapping blades [7,8].

In the context of an increasing sustainability, the possibilities offered by alkali-activation (AA) are gaining relevance. Alkali-activated materials (AAMs) are alternative cementitious materials that result from the chemical reaction between silica and alumina-rich sources and strong alkali solutions [9,10,11,12]. The alkali solutions are composed of hydroxides or silicates, or by a combination of both. The most commonly used activators are potassium or sodium hydroxide and sodium silicate. The reaction can conceptually be split into three main steps, which take place almost contemporarily: dissolution, condensation and reorganization [13,14]. During the first stage, the alkali media induces the leaching of the aluminum and silicon ions contained in the precursors. These ions then interact and condensate resulting in the formation of an intermediate complex, which is often defined as a gel. Finally, with the increase in connectivity of the gel, a condensed reorganized structure is formed, typically composed of highly connected silica and alumina tetrahedra [15]. The reaction products are mainly amorphous binders, which show remarkable features in terms of mechanical strength, chemical stability, and fire resistance. A crucial fact in the reaction kinetics is determined by the precursors’ composition, and secondarily, by the type and concentration of the alkali-activators. It is proved that a higher alkali content leads to a higher reactivity of the compounds. Nonetheless, strict control needs to be exercised on the liquid to solid ratio (L/S) of the mixture, to prevent the formation of a weak structure. A reasonably low L/S ratio, combined with a curing treatment at temperatures lower than 100 °C, is likely to give the most favorable results [16,17]. AA is quite a recent research field and because many industrial by-products present chemical compositions that match the requirements of AA, a wide range of materials are currently investigated as possible precursors.

The development of lightweight AAMs is drawn from the knowledge of cellular cement. Voids inside the cementitious binder can either be produced by adding a pre-foamed foam to the paste or by using chemical blowing agents, which release gas during the mixing phase. The addition of reactive metal powders, such as aluminum powder, liberates hydrogen gas, while a blowing agent such as hydrogen peroxide exhibits the expansion through the release of oxygen gas. The foaming technique and the mixture composition, as well as the curing conditions, differently influence the porosity and strength of the foamed materials. The combination of the alkali concentration and the L/S ratio determine both the reaction rate and the viscosity of the AA paste, which are fundamental for a stable foaming. Experience on the expansion through hydrogen peroxide showed that a correct manipulation of the AA mix permits the generation of a controlled and stable foaming. Attempts in the production of AA LWAs comprise techniques such as the granulation of hardened AAMs [18] or the cold-bonding pelletization process [19]. More recently, favorable results were achieved using a high-shear granulator, where the AA mass is mixed inside the rotating device resulting in porous granules of different grain sizes [20,21].

In this study, hydrogen peroxide is used to produce AA LWAs, which are obtained from the alkali-activation of two different waste powders, originated from different industrial processes, combined to a certain amount of metakaolin. The activator is a specific solution of sodium hydroxide and sodium silicate.

Furthermore, and likely for the first time, LWAs materials were also studied with the Time Domain (TD) Nuclear Magnetic Resonance (NMR) Relaxometry of protons (^1^H TD-MRR). This technique is an important non-destructive and non-invasive tool for analyzing the structure of porous media, ranging from biological systems [22] to cement [23]. TD-MRR is characterized by two relaxation parameters, the longitudinal relaxation time (T_1_), the transverse relaxation time (T_2_) and by a magnetization vector (the sum of the polarized nuclear magnetic moment associated to the ^1^H spins). Since a permeable porous medium is made of a network of interconnected pores of different shapes and sizes, when the TD-MRR experiment is performed on porous media fully saturated with water (or other fluid containing ^1^H as for example oil) the relaxation times will be influenced by the presence of the pore walls. Because of the auto-diffusion, a molecule of the saturating fluid continuously moves in the pore network and hits the pore walls, and so the NMR magnetization intensity relaxes back to the equilibrium over a region that is larger than a single pore (the so-called diffusion cell) [24]. TD-MRR data will therefore be averaged over the diffusion cell giving a local average information on pores. If the molecular diffusion is fast enough to maintain the magnetization uniform within the diffusion cell, then T_1_ and T_2_ show distributions of relaxation times that could be related to the distribution of pore sizes (PSD).

To obtain the PSD of a sample via TD-MRR, the surface relaxivity of the sample should be known. Many techniques can be used to obtain it, each one with its own advantages and disadvantages. Here we used the comparison with the porosity analysis carried out with the classical Mercury Intrusion Porosimetry (MIP) method. Numerous experiments have shown that in many cases the T_1_ or T_2_ relaxation time distribution curves could be very similar to the pore size distribution curves obtained by MIP [25].

Results demonstrate a significant correlation between the data obtained with NMR and the outcomes from MIP analysis.

This study proposes a new procedure to produce LWAs by AA of two different waste powders. An in-depth analysis on LWAs’ air voids content and porosity was also carried out by the means of MIP and NMR, providing practitioners and researchers with an alternative methodology to assess these characteristics.

## 2. Materials and Experimental Program

The AA process involves specific precursors and activators in a chemical reaction that leads to the development of cementing materials. In the present work, two different waste materials were used as precursors: a digested spent bentonite clay (named Ud) and a basalt powder (named B). These waste compounds were mixed with metakaolin (M) to ensure adequate chemical properties and mechanical performances to the final mix. The liquid compounds needed for the AA process were sodium silicate (SS) and sodium hydroxide (SH), according to a specific mix-design. A commercial LWA (named LECA) was used as reference material through the research.

### 2.1. Precursors

#### 2.1.1. Digested Spent Bentonite Clay

Ud used in the presented research is a waste from the food industry. The original material is a bleaching clay, which, thanks to its properties of capturing impurities from oils, is normally used for the decoloring process of oils. As a result of anaerobic digestion for biogas production, the waste materials is disposed of in a dump, with a residual oil content up to 1% by the weight of particles. After some preliminary experimental applications made by the authors for the production of AAMs with Ud, it was found that initial calcination was helping the AA reactions. In fact, the heat treatment allows the material to better interact with the activators for the AA process.

#### 2.1.2. Basalt Powder

The basalt powder is a residual from the extractions and productions processes in basalt quarries. Basalt deposits are present in almost every country and this material is widely used in the constructions field for its mineralogical, chemical, and physical properties. The extensive use of this material for bituminous mixtures and concretes leads to the production of huge quantities of powder during the preparation of aggregates. Different studies and researches have been done for the recycling and reuse of this waste powder in replacement of raw aggregates for the production of construction materials [26]. Some researchers have also verified the AA of the basalt powder, with positive outcomes [27].

#### 2.1.3. Metakaolin

The use of metakaolin (MK) to produce AAMs is common. The chemical properties of this material makes it extremely suitable for the alkali reaction process, as verified by several applications. Metakaolin is a dehydroxylated form of the clay mineral kaolinite and it is obtained by heat treatment (around 700 °C) of a natural kaolin [28].

Table 2 summarizes the precursors’ physical properties, while Table 3 shows their chemical composition.

According to scientific bibliography, the chemical composition of basalt powder is particularly suitable for its alkali-activation, as verified during the mechanical characterization of the AAMs.

### 2.2. Activators

#### 2.2.1. Sodium Silicate

SS (Na_2_SiO_3_) also commonly named as waterglass, is an aqueous solution of sodium oxide (Na_2_O) and silicon dioxide (SiO_2_) mixed according to specific proportions. Modifying the ratio between SiO_2_ and Na_2_O one can obtain a solution with different properties, which is suitable for several applications, from the construction to the food field. The SS employed in this experimentation is a commercial product, with a SiO_2_/Na_2_O ratio of 1.99 and a viscosity of 150–250 MPa·s at 20 °C.

#### 2.2.2. Sodium Hydroxide

SH (NaOH) is a solution employed to dissolve aluminosilicate, increase the pH and to compensate the electric charge of the aluminates in the mixture. It is an inorganic compound that is a highly caustic base and highly soluble in water. A 10 M SH was used in the present work. According to scientific bibliography, the SH suggested molarity for AAMs, ranges between 8 and 12 M [29,30].

### 2.3. Foaming Agent

Hydrogen peroxide (HP) was used as chemical foaming agent in this research. The HP reacts to produce oxygen gas, and the expansion process of the AAMs’ paste is due to the bubbles of O_2_ that are trapped into the mixture. It can be directly added to the AA mixture before curing, immediately developing the foaming process due to its sudden decomposition into water and oxygen gas. In this research, an HP solution 30% *w*/*w* (110 vol.) was adopted.

### 2.4. Experimental Program and Methods

The experimental program was divided into three main steps. The first one was related to the alkali-activation synthesis and to the mechanical characterization of the AAMs. In this step, no foaming agent was added to the mixtures, which were characterized by their workability and compressive strength resistance [31]. The second phase of the research program was related to the production of LWAs. The correct amount of HP needed for the expansion process was evaluated and at the same time the mixing, casting, and curing procedures of the LWAs were defined. The third step focused on the LWAs characterization. The EN 13055-1 standard [1] specifies the properties of light aggregates either obtained through natural processes or artificially produced from natural or recycled materials, used in concrete, mortar and grout in buildings, roads, and other civil engineering applications. A set of tests was selected to investigate the most relevant properties of the LWAs, with the aim of evaluating their suitability to be used as construction materials. Different tests were carried out to characterize the alkali-activated LWAs. The geometrical properties were assessed based on the particle size distribution [32]. The physical characteristics were evaluated according to the loose bulk density [33], the water content [34] and the particle density and water absorption tests [35]. According to each reference standard, a specific number of samples was tested to have significant results.

A detailed analysis of porosity and pore size distribution of LWAs were also conducted by means of both MIP and NMR techniques.

The preparation of samples measured with TD-MRR was conducted as follows. The LWAs were dried in an oven at 60 °C for 8 h and weighted (dry weight). Then the dried samples were saturated under vacuum with fresh water and weighted (saturated weight). To remove liquid from the outer surface of the aggregates (extrapellet liquid), shortly before TD-MRR analysis the LWA were removed from the liquid and rolled over a pre-soaked filter paper that ensures the liquid is not removed from the pores during the process. With the saturated and dry weights, it is possible to classically evaluate the quantity of absorbed water and therefore roughly check the quality of TD-NMR analysis. In fact, the intensity of the acquired TD-MRR signal is proportional to the amount of saturating water.

A few 20 mm internal-diameter test tubes were filled to a height of 30 mm (sensitive volume of the NMR probe) with the saturated LWAs and sealed with parafilm. TD-MRR relaxation signal curves were acquired with a console and a probe (equipped with a 25 mm internal-diameter coil) both manufactured by Stelar (Mede, PV, Italy), and using a permanent 0.18 T magnet (ESAOTE SpA, Genova, Italy).

Standard Inversion-Recovery (IR) and Carl-Purcell-Meiboom-Gill (CPMG) sequences [36] were used to acquire the T_1_ and T_2_ relaxation decay curves, respectively.

T_1_ and T_2_ relaxation decay curves were inverted to produce quasi-continuous relaxation time distributions by means of UpenWin (http://software.dicam.unibo.it/upenwin) a software that implements the 1D version of the inversion algorithm Upen [37]. Upen was specifically designed not to provide distribution details that are not supported by data, which might be misinterpreted, for example, as physically meaningful resolved pore compartments. To synthesize a complex distribution of values with only one single more manageable value, various scalar parameters (as for example, different kind of averages) could be computed from the relaxation time distribution itself. Distribution peaks position (T_(1,2)pk_) and geometric weighted average (T_(1,2)g_) are among these.

MIP experiments were performed by means of the mercury porosimeters PASCAL 140, measuring range 3.8 µm–116 µm, and PASCAL 240, measuring range 7.4 nm–15 µm (ThermoFisher Scientific, Waltham, Massachusetts, USA), where 5 g of the samples were measured using a mercury pressure range from 0 to 200 bar. The MIP data were analyzed by means of the SOL.I.D software (1.3.3, ThermoFisher Scientific, Waltham, Massachusetts, USA).

The results obtained during the characterization of the experimental aggregates, were always compared to values refer to traditional LECA.

## 3. Lightweight Aggregate Characterization

### 3.1. Alkali-Activated Material Characterization

Two different mixtures were studied using the waste materials as precursors, combined with MK according to several proportions, to achieve adequate mechanical properties. In the mix-design step, different mixes were prepared varying the following variables:

- Ud/MK and B/MK;
- SS/SH;
- Activators/Precursors;
- Curing method.

The correct mix designs and curing method were chosen according to the workability of the alkali-activated pastes and the compressive strength of the cured mixtures. In the light of the above, the optimized mix designs for the two AAM are presented in Table 4 (precursors are in percentages on the weight of the mixtures).

**Figure 1:**
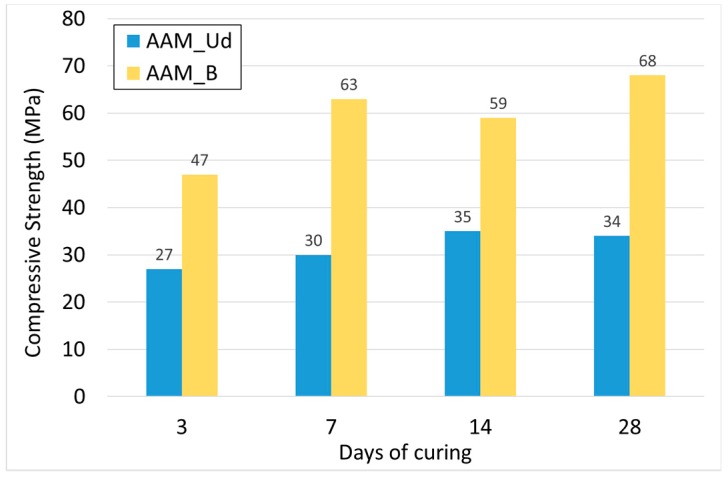
Compressive strength average results for AAM_Ud and AAM_B after 3, 7, 14 and 28 days of curing.

The higher amount of MK in the AAM_Ud is adopted to achieve consistent compressive strengths. As mentioned before, the chemical composition of B makes this waste material suitable for the alkali activation process. The presence of MK is needed to optimize the mechanical properties of the AAM_B. Both mixtures had the same workability and the different A/P ratio is mainly due the different absorption power of the mix of precursors. The performances in terms of compressive strength of both mixtures are shown in Figure 1 (avg. values of three samples). The tests were carried out on cubic samples (40 × 40 mm) initially cured for 12 h in the oven at 70 °C and then kept at ambient temperature and humidity in the laboratory.

The different mechanical resistance of the two AAMs is clear. Despite the higher presence of MK into the AAM_Ud, the compressive strength for this mixture is almost half of the AAM_B one. This is mainly due to the chemical composition of Ud, which has an Al_2_O_3_ content lower than basalt. According to scientific literature, a considerable amount of Al_2_O_3_ and SiO_2_ is favorable for the formation of a resistant microstructure during the AA process. As an overall result, it is worth noting that both mixtures reached significant compressive strength levels, if compared to other AAMs tested in several experimental applications [38,39,40,41,42] or traditional construction materials (cement concretes).

### 3.2. LWA Synthesis and Production

LWA synthesis starts from the addition of the expansion agent to the mixture after the mixing of precursors and activators for 10 min. The quantity of HP to be added to the AAMs was chosen to obtain a high workability and a good level of expansion. A specific amount of HP was added to each mixture, due to the different composition of the two experimental AAMs. Several LWAs were produced with a HP concentration between 2% and 10% by the weight of the activators. As soon as the expansion process starts, the alkali-activated samples are produced by extruding the paste from a syringe and rolling the material to form the aggregate. After being placed at 70 °C for 12 h, the samples were weighted and crushed for a preliminary analysis of their mechanical resistance.

Table 5 shows the final mix design of both mixtures, in which the amount of HP is expressed in percentage on the weight of the activators. For the LWA_Ud, the quantity of MK was also increased from the original mix design to achieve a higher mechanical strength.

**Figure 2:**
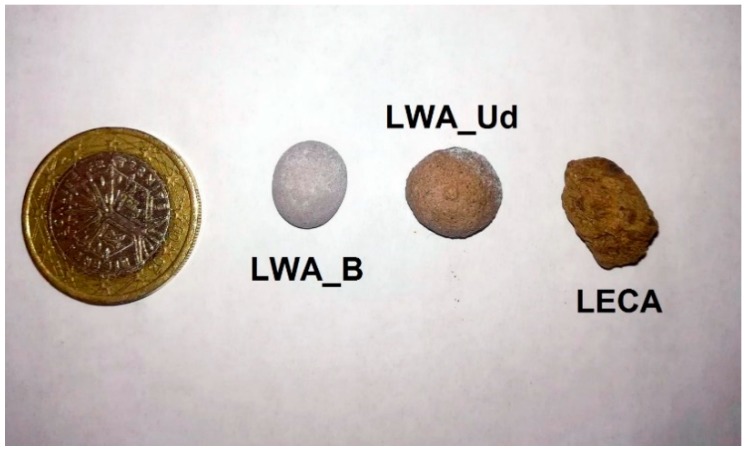
External shape of LWA_B (**left**), LWA_Ud (**center**) and LECA (**right**).

**Figure 3:**
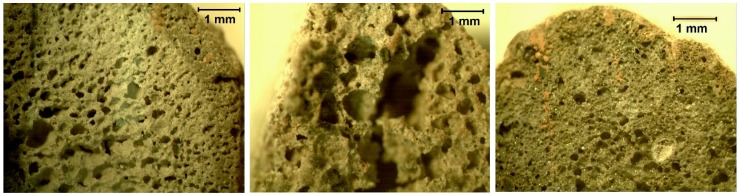
Inner structure of LWA_B (**left**), LWA_Ud (**center**) and LECA (**right**) by optical microscope.

Figure 2 and Figure 3 show the external and internal structure of the experimental LWAs and of the LECA one.

The difference in terms of external shapes between the three samples is due to the different production methods: while the experimental LWAs are handcrafted in this preliminary research stage, the LECA is produced by heat treatment. This makes the external shape irregular and the inner structure rich in small voids. From the comparison of the pictures in Figure 3, it is almost clear the difference in terms of voids between the three different samples. The LWA_Ud seems to have the largest and irregular pores, while the LECA sample shows small and well distributed voids, as confirmed by the porosity analysis shown below.

### 3.3. LWAs’ Geometrical and Physical Properties

The third stage of the research program is based on the LWA characterization. To evaluate the geometrical, physical, and mechanical characteristics of the experimental materials, laboratory tests were performed on samples in compliance with the EN 13055-1 standard [1]. The physical properties of the experimental LWAs and the reference one were assessed through the following tests:

- Particle size distribution [32];
- Loose bulk density and air voids [33];
- Water content [34];
- Particle density and Water absorption [35].

The physical analysis of LWAs was corroborated by means of NMR and MIP tests.

#### 3.3.1. Particle Size Distribution

**Figure 4:**
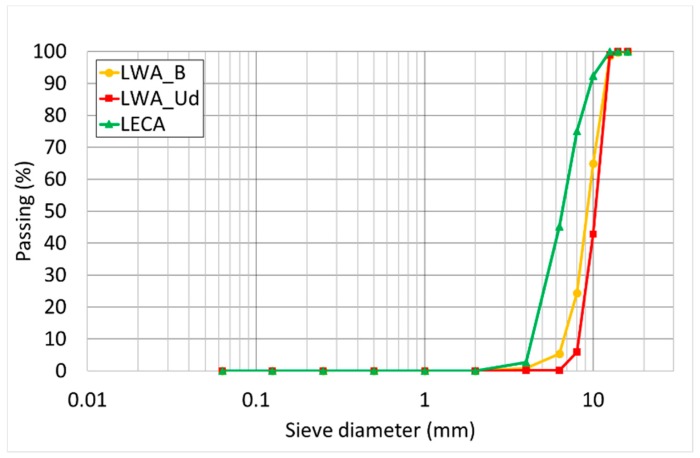
LWAs size distributions.

The determination of the particle size distribution of aggregates follows the EN 933-1 standard [32]. The test consists of dividing and separating the material into several particle size classifications of decreasing sizes by means of a series of sieves. The particle size distribution test is applied to every aggregate, including lightweight ones. Figure 4 shows the grading distributions of the three LWAs.

The particle sizes of the experimental aggregates are similar and can be classified as a 4/12.5 mm designation. The LECA grading distribution, even if classified according to the same class, is characterized by the presence of finer particles (Table 6).

#### 3.3.2. Loose Bulk Density and Air Voids 

According to the EN 1097-3 standard [33], the loose bulk density calculated as the ratio between the mass of dry aggregates filling a specific container without compaction, and the capacity of that container. From the calculation of the loose bulk density, the air voids content is evaluated as the air-filled space between the aggregates filling the container. In compliance with the standard, a 5 L container was used considering the particle size distributions of the LWAs. Results are presented in Table 7.

The loose bulk density for the LWA produced with basalt is higher if compared to the other LWAs, and this is due to the higher mass of the samples. It is worth noting that the EN 13055-1 standard [1] classifies as lightweight the aggregates with a loose bulk density not exceeding 1.20 Mg/m^3^. There is not significant difference between LWA_Ud and LECA. If the air voids are taken into account, the different results are mainly related to the grading curve of each material and the related particles shape. The standard does not specify any limits or range of values for this parameter.

#### 3.3.3. Water Content 

According to the EN 1097-5 standard [34], the water content of aggregates is evaluated by successive weighing of samples placed in a ventilated oven (110 ± 5 °C) until a constant mass is reached. The water content is determined as the difference between the wet and the dry mass and it is expressed as a percentage of the dry mass of the test portion. Table 8 shows the results.

The water content varies from 4.27% of LWA_Ud to 0.18% measured for the LECA samples. The results are highly influenced by the storing condition of the samples. All the material were kept at ambient temperature inside the laboratory, stored in plastic bags. Both tests on basalt and expanded clay aggregates were performed during a dry season, whereas the tests on Ud specimens were performed when climate conditions were more humid. In the light of the above, further test are needed to verify the water content of LWAs on similar storing conditions. It shall be mentioned that EN 13055-1 [1] does not specify any water content limit for the LWAs.

#### 3.3.4. Particle Density and Water Absorption 

The EN 1097-6 standard [35] describes the pycnometer method for the evaluation of three different density parameters and the water absorption values for LWAs. According to the standard, the particle density is given by the ratio of mass to volume. Based on the conditions of weighting the test portion, the density is considered in saturated, saturated, and surface-dried (SSD) and in the oven-dried condition. The water absorption is then calculated using a soaking time of 24 h. Average results are presented in Table 9.

The densities of the three LWAs are different because of the inner structure given by the expansion process. LECA is the lightest material while LWA_B is the heaviest one. It is worth noting that according to the EN 13055-1 standard [1] the aggregates with a particle density not exceeding 2.00 Mg/m^3^ are classified as lightweight. The water absorption values are strictly connected to the pores dimensions. Further observations on particle densities and porosity are shown in the porosity analysis section of this paper.

#### 3.3.5. Analysis of Porosity

To obtain the relaxation time distributions, the relaxation decay curves were inverted by means of UpenWin.

**Figure 5:**
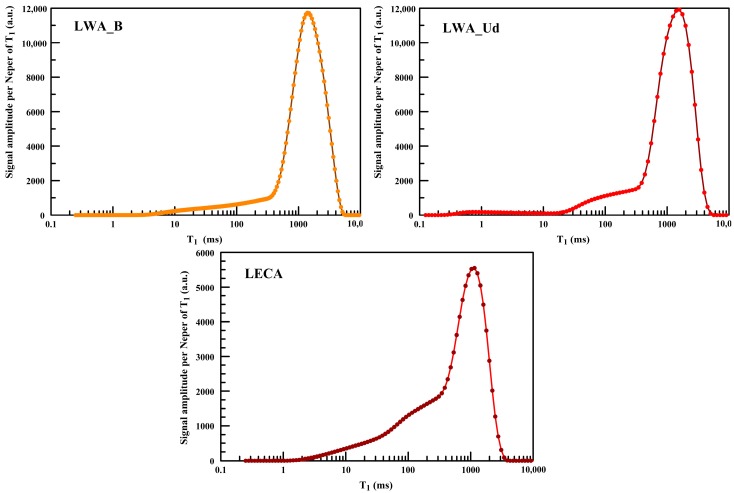
T_1_ distributions for LWA_B, LWA_Ud and LECA.

At first sight, the T_2_ distributions (not shown) encoded at different echo time, allow us to state that there are not evident diffusion effects, more precisely, the T_2_ distributions do not change varying the echo time. Therefore, the relaxation time distributions are strictly related to the local diffusion cell and consequently they are representative of the PSD. In Figure 5 the T_1_ distributions of samples are shown. All the distributions show a well-defined and narrow peak at long times (around 1 s) followed by long tail with a hump at shorter times. The areas under the relaxation curves are proportional to the NMR signals which, in turn, are proportional to the amount of saturating water.

The T_1pk_ of the three distributions are respectively: 1108, 1417 and 1519 milliseconds. Table 10 summarizes the weights and the NMR signal intensity computed on the distributions. The good proportionality of column 4 and 5, the maximum percentage discrepancy of their ratio (column 6) is approximately 7%, allows us to state that the TD-MRR measurements were significant and reliable.

**Figure 6:**
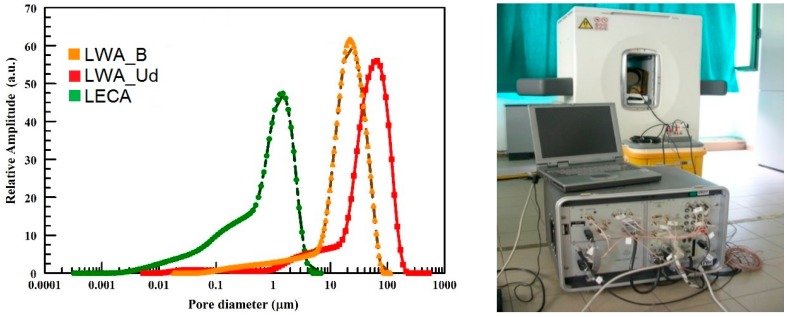
TD-MRR PSDs for LWA_B, LWA_Ud and LECA (**left**) and NMR equipment (**right**).

The TD-MRR PSDs obtained by calibrating the TD-MRR relaxation time distributions with MIP results are shown in Figure 6. The samples have significantly different PSDs. In particular, small pores in the range from few nanometers to some micrometers characterize the LECA aggregates. LWA_B and LWA_Ud have larger pores in the range of 0.1–200 mm. If the two experimental aggregates are compared, the LWA_Ud has the larger pores, with a consistent density of pores whose size ranges between 100 and 200 mm. Moreover, form the analysis of the area under the distribution curves, both experimental aggregates are characterized by a more homogeneous pore size distribution. These results are in line with that verified from the optical microscope images shown in Figure 3. The inner structure of LECA consists of small pores if compared to the experimental aggregates, while the Ud one has the largest diameter voids.

### 3.4. LWAs’ Mechanical Properties

The mechanical characterization of LWAs is made through the evaluation of their crushing resistance. The EN 13055-1 standard [1] specifies the apparatus and two different test procedures related the loose bulk density of the LWAs. Based on previous results, the procedure n.2 was adopted. According to the standard, three test specimens were prepared by filling a specific steel cylinder with aggregates. When compacted, the LWAs surface was leveled to the top rim of the container, which was then subject to a force given by a piston set to reach a compression of 20 mm in approximately 100 s. The crushing resistance for each test specimen was than calculated using an equation that considers the load exerted by the piston, its area and the compression force and the area. Average results are shown in Table 11.

Comparing the results, LWA_B shows the highest mechanical performance: its crushing resistance is three times that of LWA_Ud. If LECA is taken into account, the increase in crushing resistance for LWA_B is about 34%. As general remark, it must be noted that the standard does not specify any limits or range of results in terms of crushing resistance for LWAs. However, considering the wide field of applications of these materials, a minimum value of crushing resistance is required for LWAs to be used as construction materials. Generally, according to the different uses in the civil engineering field, the threshold limit is set at 0.7 N/mm^2^. As an example, for foundation layers between the floor and the finishing flooring, for the thermal and acoustic insulation of a building, a crushing resistance between 0.7 and 1.5 is generally required. For structural lightweight concrete, this range is raised to a minimum of 4.5 N/mm^2^ while for lightweight asphalt concretes the Italian technical specification requires a crushing resistance higher than 2.7 N/mm^2^. In the light of the above, both the experimental LWAs meet the required minimum mechanical properties for their applications in the civil engineering field.

## 4. Conclusions

The study presented in the paper shows a new method to produce alternative LWAs by AA of two different waste powders. Two experimental alkali-activated LWAs have been developed using a basalt powder and a digested spent bentonite clay as main precursors. Based on the data presented in this work, several conclusions can be drawn which corroborate the validity and the smartness of the proposed solution for the production of alternative LWAs:

- The adopted waste materials were suitable for the AA process. The addition of HP to the pastes allowed the production of lightweight aggregates in the laboratory. The optimum quantity of the foaming agent was determined to obtain a workable paste and final aggregates with a good porosity-to-strength ratio.
- Because of the physical characterization of the LWAs, both the experimental materials showed appropriate properties to be classified as lightweight aggregates according to the EN 13055-1 standard [1]. LWA_B and LWA_Ud have densities lower than 2.00 Mg/m^3^ and a loose bulk density not exceeding 1.20 Mg/m^3^.
- Mechanical characterization was performed through the crushing resistance test. Even if the EN 13055-1 [1] does not specify any limits for LWAs, a specific crushing resistance value is required for their application as construction material. LWA_Ud has a crushing resistance (1.07 N/mm^2^) higher than the lower limit usually specified for the application of LWAs in civil engineering projects. LWA_B has the highest mechanical resistance (4.44 N/mm^2^) even if compared to the structural expanded clay, LECA, used in this research as reference lightweight construction material. These results are in line with the values recorded during the preliminary mechanical characterization of the alkali-activated paste materials and are mainly dependent on the chemical composition of the wastes used as precursors.
- The voids of the LWAs materials were studied by means of the Time Domain NMR Relaxometry of protons (^1^H TD-MRR). The TD-MRR results were then compared with the porosity analysis carried out with the MIP method. In this work, the relaxation time distributions are strictly related to the local diffusion cell and effectively represent the distribution of pore sizes (PSD). From the correlation with MIP results, the TD-MRR PSD shows a structure rich in small voids for LECA samples, while larger and more size-homogeneous pores characterize the experimental aggregates. These results are corroborated by the visual analysis of the optical microscope images, which confirm the presence of larger voids for LWA_Ud if compared to LWA_B and LECA. Small pores instead characterize the latter.

In light of the above, the expansion of these AA waste materials seems to be a viable solution for the production of lightweight aggregates, allowing the recycling of materials otherwise dumped or sent to landfill. Moreover, the results confirm and validate the analysis of pore sizes and their distribution in lightweight aggregates according to TD-MRR and MIP methodologies.

Further investigations and tests are planned to back up these preliminary conclusions and to confirm the use of AA LWAs as construction materials. Additional research will focus on the Life Cycle Analysis of the proposed solution to justify their development on an industrial scale.

## Figures and Tables

**Table 1:** Table of notations.

Notation	Description
LWA	Light weight aggregate
LECA	Lightweight expanded clay aggregate
AAM	Alkali-activated material
L/S	Liquid-solid ratio
NMR	Nuclear Magnetic Resonance
TD-MRR	Time Domain Nuclear Magnetic Resonance Relaxometry
PSD	Distribution of pore sizes
MIP	Mercury Intrusion Porosimetry
Ud	Digested spent bentonite clay
B	Basalt powder
MK	Metakaolin
SS	Sodium silicate
SH	Sodium hydroxide
HP	Hydrogen peroxide
A/P	Activator-precursor ratio
IR	Standard Inversion-Recovery
CPMG	Carl-Purcell-Meiboom-Gill sequence
SSD	Saturated and surface-dried

**Table 2:** Ud, B and M physical properties.

Test	Unit	Ud	B	MK
Size distribution (EN 13043)	%	100 P_50um_	100 P_50um_	95 P_80um_
Water content (EN 1097-5)	%	1.12	0.04	0.12
Particle density (EN 1097-7)	Mg/m^3^	1.86	2.70	2.40

**Table 3:** Precursors’ chemical composition.

Compound	Unit	Ud	B	MK
SiO_2_	% p/p	43.9	45.3	55.2
CaO	% p/p	2.2	8.8	0.2
Na_2_O	% p/p	1.2	1.7	0.6
Al_2_O_3_	% p/p	9.7	21.6	40.3
Fe_2_O_3_	% p/p	5.4	8.5	1.4
SO_3_	% p/p	1.4	<0.1	0.2
MgO	% p/p	5.7	2.0	0.1
P_2_O_5_	% p/p	0.7	0.7	<0.1
TiO_2_	% p/p	0.8	0.2	1.5
ZnO	% p/p	<0.1	<0.1	<0.1
K_2_O	% p/p	0.8	9.7	0.2

**Table 4:** Mix design for AAM_Ud and AAM_B.

Mixture	Ud (%)	B (%)	MK (%)	SS/SH	A/P
AAM_Ud	50	0	50	3	1
AAM_B	0	70	30	4	0.45

**Table 5:** Mix design for LWA_Ud and LWA_B.

Mixture	Ud (%)	B (%)	MK (%)	SS/SH	A/P	HP (%)
LWA_Ud	40	0	60	3	1	5
LWA_B	0	70	30	4	0.45	7

**Table 6:** Passing material for LWA_B, LWA_Ud and LECA.

Sieve (mm)	Passing LWA_B (%)	Passing LWA_Ud (%)	Passing LECA (%)
16	100.00	100.00	100.00
14	99.71	100.00	100.00
12.5	98.57	98.99	100.00
10	64.90	42.84	92.39
8	24.40	5.88	75.04
6.3	5.39	0.17	45.11
4	0.86	0.06	2.64
1	0.00	0.00	0.00
0.5	0.00	0.00	0.00
0.063	0.00	0.00	0.00

**Table 7:** Loose bulk densities and voids content for the LWAs.

	LWA_B	LWA_Ud	LECA
L.b.d.—Sample 1 (Mg/m^3^)	0.701	0.473	0.422
L.b.d.—Sample 2 (Mg/m^3^)	0.703	0.476	0.420
L.b.d.—Sample 3 (Mg/m^3^)	0.702	0.475	0.424
Avg. L.b.d. (Mg/m^3^)	0.702	0.475	0.422
Avg. Air voids (%)	44.3	36.0	43.5

**Table 8:** Water content for the LWAs.

	LWA_B	LWA_Ud	LECA
Water content (%)—Sample 1	1.27	4.10	0.19
Water content (%)—Sample 2	1.27	4.44	0.17
Avg. Water content (%)	1.27	4.27	0.18

**Table 9:** Apparent, oven-dried, SSD particle densities and water absorption values for LWAs.

	LWA_B	LWA_Ud	LECA
Apparent particles density (Mg/m^3^)	1.69	1.18	0.87
Oven-dried particles density (Mg/m^3^)	1.26	0.74	0.75
SSD particles density (Mg/m^3^)	1.52	1.11	0.85
Water absorption after 24 h (%)	20	50	17

**Table 10:** Weight of the samples used for the NMR measurements and the total NMR signal of the T_1_ distributions.

Sample	Dry Weight (g)	Saturated Weight (g)	Absorbed Water (g)	T1 Total Signal (Arbitrary Unit)	Ratio [-]
LWA_B	5.7	8.9	3.2	19,050	5953
LWA_Ud	4.1	7.7	3.6	21,290	5914
LECA	4.4	6.5	2.1	11,690	5567

**Table 11:** Compression force and crushing resistance for LWA_B, LWA_Ud and LECA.

	LWA_B	LWA_Ud	LECA
Compression force (N)	51,100	12,150	38,050
Crushing resistance (N/mm^2^)	4.44	1.07	3.31
